# Plasma adiponectin, visfatin, leptin, and resistin levels and the onset of colonic polyps in patients with prediabetes

**DOI:** 10.1186/s12902-020-0540-7

**Published:** 2020-05-11

**Authors:** Lili Deng, Xiaotong Zhao, Mingwei Chen, Hua Ji, Qunhui Zhang, Ruofei Chen, Yalei Wang

**Affiliations:** 1 Department of Endocrinology, the First Affiliated Hospital of Anhui Medical University, No.218 Jixi Road, Hefei, 230032 Anhui Province China; 2 Institute of Traditional Chinese Medicine for the Prevention and Control of Diabetes, Anhui Academy of Chinese Medicine, Hefei, 230032 Anhui Province China; 3 Anhui Medical University Clinical College, No.81 Meishan Road, Hefei, 230032 Anhui Province China; 4 Department of Gastroenterology, the First Affiliated Hospital of Anhui Medical University, No.218 Jixi Road, Hefei, 230032 Anhui Province China

**Keywords:** Prediabetes, Colonic polyps, Adipokine, Risk factors

## Abstract

**Background:**

Prediabetes is associated with a high risk of colon cancer, and abdominal obesity, which can result in the secretion of several obesity-related adipocytokines, is an independent influencing factor for colonic polyps in prediabetes subjects. However, the correlation between adipocytokine levels and colonic polyps in prediabetes subjects is unclear. This research explores the relationship between plasma adiponectin, visfatin, leptin, and resistin levels and the development of colonic polyps in prediabetes subjects.

**Methods:**

A total of 468 prediabetes subjects who underwent electronic colonoscopy examinations were enrolled in this study; there were 248 cases of colonic polyps and 220 cases without colonic mucosal lesions. Then, colonic polyps patients with prediabetes were subdivided into a single-polyp group, multiple-polyps group, low-risk polyps group, or high-risk polyps group. In addition, 108 subjects with normal glucose tolerance who were frequency matched with prediabetes subjects by sex and age were selected as the control group; 46 control subjects had polyps, and 62 control subjects were polyp-free. Plasma adiponectin, visfatin, leptin, and resistin levels were measured in all the subjects, and the related risk factors of colonic polyps in prediabetes subjects were analysed.

**Results:**

Plasma adiponectin levels were significantly lower in the polyps group than in the polyp-free group [normal glucose tolerance (9.8 ± 4.8 vs 13.3 ± 3.9) mg/L, *P* = 0.013; prediabetes (5.6 ± 3.7 vs 9.2 ± 4.4) mg/L, *P* = 0.007]. In prediabetes subjects, plasma adiponectin levels were decreased significantly in the multiple polyps group [(4.3 ± 2.6 vs 6.7 ± 3.9) mg/L, *P* = 0.031] and the high-risk polyps group [(3.7 ± 2.9 vs 7.4 ± 3.5) mg/L, *P* < 0.001] compared to their control groups. Plasma visfatin levels were higher in the polyps group and the multiple-polyps group than those in their control groups (*P* = 0.041 and 0.042, respectively), and no significant difference in plasma leptin and resistin levels was observed between these three pairs of groups (all *P* > 0.05). The multivariate logistic regression analysis showed that lower levels of plasma adiponectin was a risk factor for colonic polyps, multiple colonic polyps, and high-risk colonic polyps in prediabetes subjects.

**Conclusions:**

Plasma adiponectin levels are inversely associated with colonic polyps, multiple colonic polyps, and high-risk colonic polyps in prediabetes subjects. And adiponectin may be involved in the development of colon tumours in prediabetes subjects.

## Background

Prediabetes is a hyperglycaemic state between normal glucose tolerance and diabetes and is recognized as an important risk factor for the development of diabetes [1]. Prediabetes can lead to an increased risk of various forms of cardiovascular disease, which has become the leading cause of mortality in type 2 diabetes [2, 3]. And diabetes and prediabetes are also associated with an increased risk of cancer-related death, and mortality increases with increasing glucose concentrations in a linear manner across all types of cancers [4]. Colorectal cancer accounts for about 10% of the total tumor incidence, ranking third and second respectively in men and women worldwide [5]. It is widely known that most colorectal cancers develope from colorectal adenomas through the adenoma-carcinoma sequence. Recently, Cha JM et al. reported that prediabetes was associated with an increased prevalence of multiple and high-risk colorectal adenomas and was an independent risk factor for high-risk colorectal adenoma [6]; however, the biological mechanism linking prediabetes and the presence of colorectal adenoma has not been fully elucidated.

The high prevalence of obesity, especially abdominal obesity, has been hypothesized to be among the factors responsible for the high incidence of colorectal cancer in most developed countries [7]. The current body of evidence points to a causal role of obesity, in particular abdominal obesity, and abnormal glucose metabolism in the development of colorectal cancer [8, 9]. Comstock SS et al. found that obese men are more likely to have at least three polyps and adenomas than non-obese men [10]. In recent years, some scholars have suggested and reported that adipokines secreted by adipose tissue may play an important role in the pathogenesis of obesity and colon cancer using several in vivo, in vitro, and epidemiological investigations [11]; the attention was mainly focused on plasma adiponectin, visfatin, leptin, and resistin [12, 13]. Until now, the correlation between plasma adiponectin, visfatin, leptin, and resistin levels and the incidence of colonic polyps in the prediabetes population, as well as the number and degree of malignant polyps in the colon, is still unclear. Actively exploring the relevant factors affecting colonic polyps in prediabetes patients is of great significance for effective colon cancer prevention and treatment and improvement in colon cancer prognosis.

In this study, we investigated whether changes in plasma adiponectin, visfatin, leptin, and resistin levels are associated with the onset of colonic polyps in prediabetes subjects. To the best of our knowledge, this is the first study to evaluate the role of adiponectin, visfatin, leptin, and resistin on the development of colonic polyps in prediabetes subjects.

## Methods

### Study design and participants

**Fig. 1:**
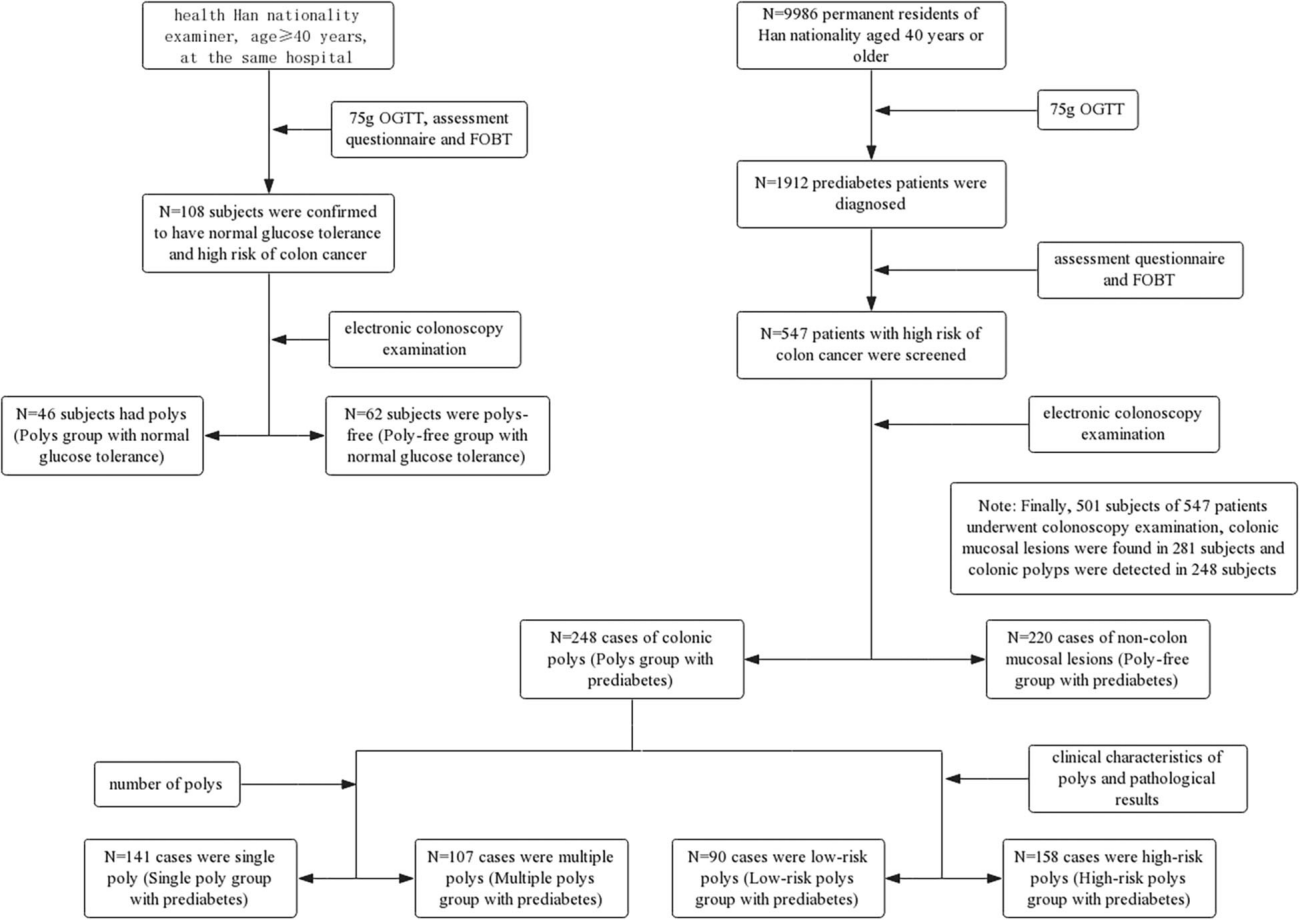
An overview of the study design and allocation of participants

Following the operation manual developed by the “Epidemiology Research on Tumor Risks in Type 2 Diabetes Patients in China (REACTION study)” group and using the cluster sampling method, from April 2012 to December 2012 we carried out a cross-sectional survey among residents who were Han (ethnicity) and aged 40 years or older living in four communities in Hefei, Anhui, China. We requested physical examinations and blood lipid and blood glucose tests to be conducted in 9986 subjects. Moreover, all the non-diabetic subjects were requested to undergo a 75 g oral glucose tolerance test (75 g OGTT); the results indicated 1912 prediabetes cases, which were considered in the survey. Then, on the basis of the provisions of the “Guidelines for Early Screening and Early Treatment of Chinese Cancer (trial edition)” which was developed by the Ministry of Health of China in 2005 and suggests a combined mathematical risk factor model assessment questionnaire and faecal occult blood test (FOBT), we evaluated the risk of colon cancer among the 1912 prediabetes subjects (excluding those who were diagnosed with colon cancer). After screening, those who met one of the following criteria were considered high risk for colon cancer: (1) those whose FOBT was positive; (2) those whose first-degree relatives had a history of colorectal cancer; (3) those who had a history of cancer or intestinal polyps; and (4) those who had two or more of the following: chronic constipation, chronic diarrhoea, mucous bloody stool, history of chronic appendicitis or appendectomy, and history of chronic cholecystitis or gallstones. This investigation was presented in detail in our previous study [14]. As a result, a total of 547 patients with high risk of colon cancer were screened, and we suggested that these patients undergo an electronic colonoscopy examination. Finally, 501 subjects underwent electronic colonoscopy examinations, colonic mucosal lesions were found in 281 subjects, and colonic polyps were detected in 248 subjects who were assigned to the polyp group with prediabetes. In addition, non-colon mucosal lesions were found in 220 subjects who were assigned to the polyp-free group with prediabetes. The total of 468 prediabetic with or without colonic polyps were the subjects of this study. (Fig. 1).

In the present study, the controls were 108 Chinese patients of Han nationality who were aged ≥40 years, who were frequency matched by sex and age with prediabetes subjects and confirmed to have normal glucose tolerance by a 75 g oral glucose tolerance test, and who agreed to a mathematical risk factor model assessment questionnaire and faecal occult blood test (FOBT) according to the “Guidelines for Early Screening and Early Treatment of Chinese Cancer (trial edition)” mentioned above during the same period and at the same hospital, with the same exclusion criteria. Then, 108 controls were received electronic colonoscopy examinations. And colonic polyps were detected in 46 subjects who were assigned to the polyp group with normal glucose tolerance. In addition, non-colon mucosal lesions were found in 62 subjects who were assigned to the polyp-free with normal glucose tolerance group (Fig. 1).

The criteria for inclusion were as follows: (1) those who were Han, aged 40 years or older, and male or female; and (2) those who provided written consent. The exclusion criteria were as follows: (1) those who received oral glucocorticoid treatment over the past 3 months; (2) those who previously had hypothalamic, pituitary, adrenal, thyroid disease; (3) those who had severe heart, liver, kidney dysfunction, or chronic wasting disease; (4) those who applied for any hypoglycaemic drugs for pre-diabetes intervention; (5) those who had taken statins, nonsteroidal anti-inflammatory analgesics, and aspirin for more than 1 month in the past 6 months; (6) those whose weight loss exceeded 5% over the past 3 months; (7) those who had inflammatory bowel disease (such as ulcerative colitis, Crohn’s disease, etc.); (8) those who had an evident diagnosis of colon cancer and colon polyp lesions; (9) those who experienced a history of gastrointestinal surgery; and (10) those who had incomplete coloscopy due to poor bowel preparation or failure to carry out caecal intubation. Prediabetes is a condition that meets the diagnostic criteria for the classification of glucose metabolism states, which were promulgated by the World Health Organization (WHO) in 1999. This study was approved by the Ethics Committee of the First Affiliated Hospital of Anhui Medical University.

### Diagnosis and classification of colonic polyps

The results of electronic colonoscopy examinations regarding colonic polyps were recorded by a single researcher. If necessary, a polyp biopsy was performed, and the specimens were sent for pathological examination. When measuring the diameter of multiple polyps, the largest number was considered. The number of polyps was divided into single polyps (number of polyps ≤2) and multiple polyps (number of polyps ≥3). According to the clinical characteristics of the polyps and the pathological results described in a previous study [6], polyps were divided into high-risk polyps (number of polyps ≥3 and/or high hyperplasia and/or villous structures and/or polyps diameter ≥ 1 cm) and low-risk polyps (number of polyps < 3 and/or without high hyperplasia and/or tubular structure and/or polyp diameter < 1 cm).

### Definition of observation indicators

Each participant was asked to answer a questionnaire covering their lifestyle and personal and family medical history. Briefly, the questionnaires mainly included the subjects’ smoking habits, alcohol drinking, physical activity, vegetable consumption, medications (such as statins, nonsteroidal anti-inflammatory analgesics, and aspirin) and family history of colon cancer. The definition of these indicators was presented as described in our previous studies [13, 15]: current smoking was referred to as smoking at least five cigarettes per day on average and lasted for one year or more. Regular alcohol drinking was referred to as drinking an average of at least 25 g per day for more than 1 year. Regular exercise was referred to as an average of five exercise events per week lasting at least 30 min per event and a total of time at least 150 min per week. Regular vegetable consumption was referred to as intaking of more than 250 g of vegetables per day. In addition, the family history of colon cancer was referred to as colon cancer in first-degree relatives of the subjects.

### Physical examination and laboratory testing

The participants fasted overnight before their endoscopic examination; then, their height, weight, waist circumference, hip circumference, systolic blood pressure (SBP), and diastolic blood pressure (DBP) were measured by a trained investigator as routine practice, and their body mass index (BMI) and waist:hip ratio (WHR) were calculated. Moreover, the collected venous blood samples were stored at − 80 °C for unified detection. Glucose, insulin, adiponectin, visfatin, leptin and resistin levels were measured in plasma samples, and total cholesterol (TCH) and triglycerides (TGs) were measured in serum samples. Insulin resistance was evaluated by homeostatic model assessment of insulin resistance (HOMA-IR) using the equation HOMA-IR = fasting glucose (mmol/L) × fasting insulin (mIU/L)/22.5.

The plasma/serum level of each index was determined as described in our previous study [13, 15]: fasting plasma glucose (FPG) and postprandial 2 h plasma glucose (2hPG) were detected by the glucose oxidase method; and TGs and TCH were measured by the enzymatic method. Fasting insulin (FINS) was measured by chemiluminescence. Plasma adiponectin, visfatin, leptin, and resistin were analysed in one run by enzyme-linked immunosorbent assay (ELISA).

### Statistical analyses

The SPSS statistical software package for Window version 17.0 (SPSS, Chicago, IL, USA) was used for all statistical analyses. The continuous variables are expressed as means±standard deviations, and the categorical variables are expressed as numbers (percentages). The *x*^2^ test and One-way analysis of variance were used for comparisons of clinical parameters among polyp-free group with normal glucose tolerance, colonic polyps group with normal glucose tolerance, polyp-free group with prediabetes, and colonic polyps group with prediabetes, and least-significant difference (LSD) analysis was used for comparisons between two groups. For comparisons between single polyp group and multiple polyps group/low-risk polyps group and high-risk polyps group in prediabetes, normally distributed continuous variables were analysed using independent sample t-tests, non-normally distributed continuous variables were analysed by Mann-Whitney U tests, and count data were analysed using the *x*^2^ test. Multivariate unconditional logistic regression was used to analyse the influencing factors of colonic polyps, including multiple colonic polyps and high-risk colonic polyps. All *P*-values are two-sided, and a value less than 0.05 was considered statistically significant.

## Results

### Comparison of clinical parameters among the polyp-free group with normal glucose tolerance, colonic polyps group with normal glucose tolerance, polyp-free group with prediabetes, and colonic polyps group with prediabetes

**Fig. 2:**
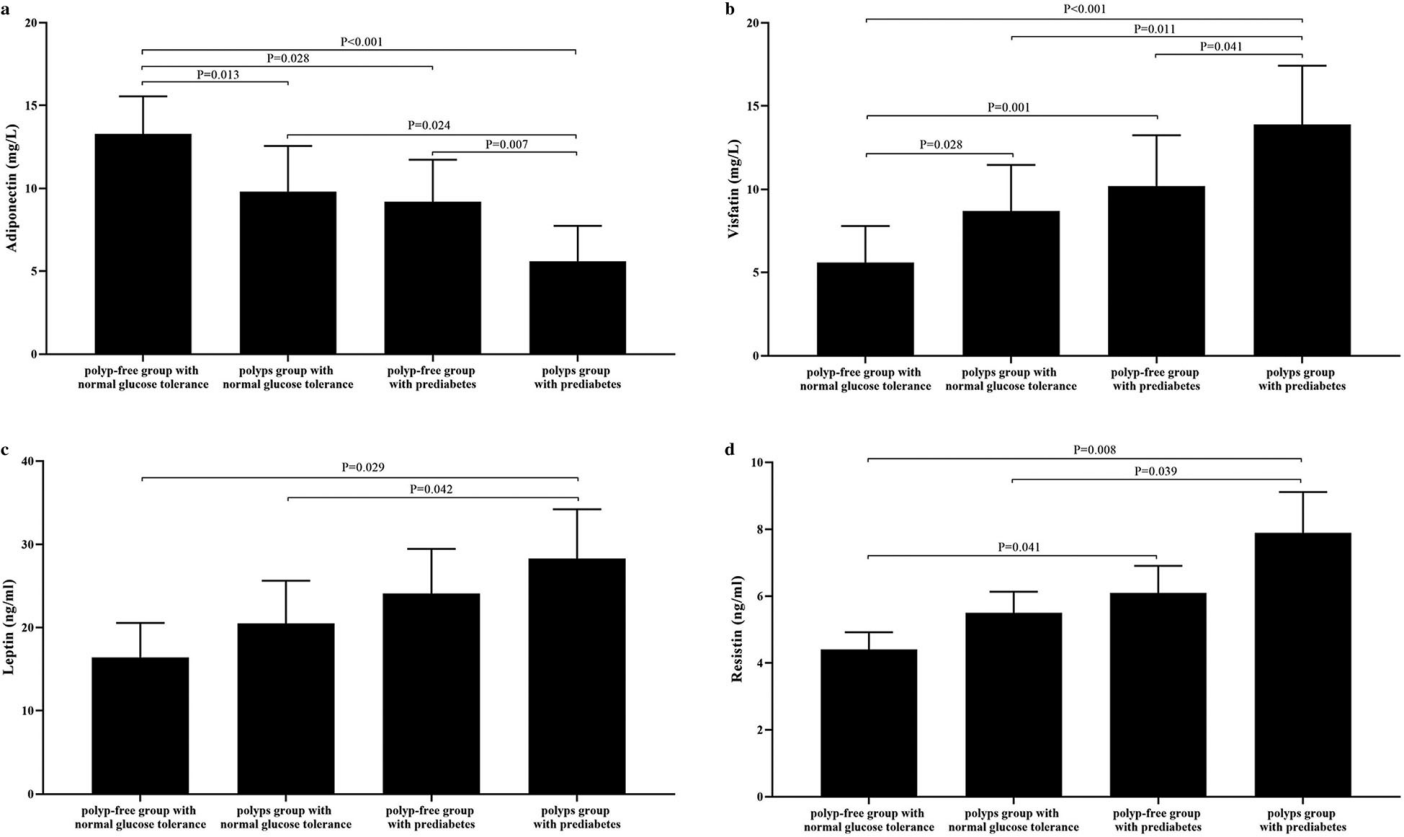
Plasma adiponectin, visfatin, leptin, and resistin levels among polyp-free group with normal glucose tolerance, polyps group with normal glucose tolerance, polyp-free group with prediabetes, and polyps group with prediabetes. The expression of (**a**) plasma adiponectin, (**b**) plasma visfatin, (**c**) plasma leptin and (**d**) plasma resistin levels among these four groups were showed on panels

Compared with the polyp-free group with normal glucose tolerance, the average age, plasma visfatin levels, and proportion of family history with colon cancer in the colonic polyps group with normal glucose tolerance were significantly higher (*P* = 0.032 for age; *P* = 0.028 for visfatin; *P* = 0.042 for family history with colon cancer, respectively); plasma adiponectin levels were significantly lower [(9.8 ± 4.8 vs 13.3 ± 3.9) mg/L, *P* = 0.013], and the other clinical parameters were not statistically significant between the two groups (*P* > 0.05) (Table 1, Fig. 2).

**Table 1:** Comparison of clinical parameters among people with normal glucose tolerance and prediabetes [±s, *n* (%)]

Variable	Polyp-free group with normal glucose tolerance (*n* = 62)	Polyps group with normal glucose tolerance (*n* = 46)	Polyp-free group with prediabetes (*n* = 220)	Polyps group with prediabetes (*n* = 248)	*P*-value
Sex (male/female)	62 (32/30)	46 (26/20)	220 (98/122)	248 (156/92) f	0.001
Age (year)	51.1 ± 11.1	54.4 ± 13.2 a	50.2 ± 10.9 c	56.3 ± 12.3 ae	0.008
BMI (kg/m^2^)	23.1 ± 2.8	23.8 ± 3.1	23.7 ± 3.4	24.9 ± 3.3 bce	0.017
WHR	0.86 ± 0.04	0.87 ± 0.05	0.88 ± 0.07	0.91 ± 0.08 ace	0.004
SBP (mm Hg)	121 ± 10	124 ± 14	124 ± 13	122 ± 14	0.264
DBP (mm Hg)	75 ± 13	77 ± 12	76 ± 12	78 ± 10	0.318
FPG (mmol/L)	4.8 ± 0.6	4.9 ± 0.5	5.8 ± 0.5 ad	5.9 ± 0.4 ad	0.029
2hPG (mmol/L)	6.7 ± 0.8	6.8 ± 0.9	8.9 ± 1.1 bd	9.9 ± 1.0 bde	< 0.001
TG (mmol/L)	1.32 ± 0.81	1.56 ± 0.83	1.54 ± 0.91	1.69 ± 0.82 ace	0.037
TCH (mmol/L)	4.32 ± 0.75	4.65 ± 0.72	4.98 ± 0.89	5.27 ± 0.68	0.261
FINS (mIU/L)	9.31 ± 4.22	12.53 ± 5.38	11.52 ± 6.27	14.71 ± 6.75 a	0.042
HOMA-IR	1.98 ± 1.47	2.73 ± 1.58	2.96 ± 1.15 a	3.86 ± 1.92 bc	0.005
Adiponectin (mg/L)	13.3 ± 3.9	9.8 ± 4.8 a	9.2 ± 4.4 a	5.6 ± 3.7 bcf	< 0.001
Visfatin (mg/L)	5.6 ± 3.8	8.7 ± 4.8 a	10.2 ± 5.3 b	13.9 ± 6.1 bce	0.007
Leptin (ng/ml)	16.4 ± 7.2	20.5 ± 8.9	24.1 ± 9.3	28.3 ± 10.2 ac	0.039
Resistin (ng/ml)	4.4 ± 0.9	5.5 ± 1.1	6.1 ± 1.4 a	7.9 ± 2.1 bc	0.041
Current smoking (%)	19 (30.6)	16 (34.8)	51 (23.2)	95 (38.3) f	0.005
Regular alcohol drinking (%)	18 (29.0)	16 (34.9)	50 (22.7)	97 (39.1) f	0.002
Regular exercise (%)	20 (32.3)	13 (28.3)	64 (29.1)	53 (21.4)	0.156
Regular vegetables consumption (%)	24 (38.7)	12 (26.1)	68 (30.9)	69 (27.8)	0.359
Family history of colon cancer (%)	2 (3.2)	6 (8.7) a	13 (5.9)	25 (10.1) be	0.005

Data are presented mean ± standard deviations or numbers (%); Differences among four groups analyzed using one-way analysis of variance or *x*^2^ test, and least-significant difference (LSD) analysis was used for comparison between the two groups. Versus polyp-free group with normal glucose tolerance, a = *P* < 0.05, b = *P* < 0.01; versus polyps group with normal glucose tolerance, c = *P* < 0.05, d = *P* < 0.01;versus polyp-free group with prediabetes, e = *P* < 0.05, f = *P* < 0.01

Compared with the polyp-free group with normal glucose tolerance, FPG, 2hPG, HOMA-IR, plasma visfatin, and resistin levels were significantly higher in the polyp-free group with prediabetes (*P* = 0.025 for FPG; *P* = 0.009 for 2hPG; *P* = 0.034 for HOMA-IR; *P* = 0.001 for visfatin; *P* = 0.041 for resistin, respectively); plasma adiponectin levels were significantly lower [(9.2 ± 4.4 vs 13.3 ± 3.9) mg/L, *P* = 0.028]; and the other clinical parameters were not statistically significant between the two groups (*P* > 0.05) (Table 1, Fig. 2).

Compared with the polyp-free group with normal glucose tolerance, the average age, BMI, WHR, FPG, 2hPG, TGs, FINS, HOMA-IR, visfatin, leptin and resistin levels, and the proportion of family history with colon cancer were significantly higher in the colonic polyps group with prediabetes (*P* = 0.017 for age; *P* = 0.006 for BMI; *P* = 0.024 for WHR; *P* = 0.011 for FPG; *P* < 0.001 for 2hPG; *P* = 0.042 for TGs; *P* = 0.047 for FINS; *P* < 0.001 for HOMA-IR; *P* < 0.001 for visfatin; *P* = 0.029 for leptin; *P* = 0.008 for resistin; *P* = 0.008 for family history with colon cancer, respectively), and the level of adiponectin was significantly lower [(5.6 ± 3.7 vs 13.3 ± 3.9) mg/L, *P* < 0.001). The other clinical parameters were not statistically significant between the two groups (*P* > 0.05) (Table 1, Fig. 2).

Compared with the colonic polyps group with normal glucose tolerance, the FPG and 2hPG levels in the polyp-free group with prediabetes were significantly higher (*P* = 0.001 for FPG; *P* < 0.001 for 2hPG, respectively), and age was lower (*P* = 0.015). The other clinical parameters were not statistically significant between the two groups (*P* > 0.05) (Table 1).

Compared with the colonic polyps group with normal glucose tolerance, the BMI, WHR, FPG, 2hPG, TGs, HOMA-IR, visfatin, leptin, and resistin levels were significantly higher in the colonic polyps group with prediabetes (*P* = 0.026 for BMI; *P* = 0.033 for WHR; *P* = 0.001 for FPG; *P* < 0.001 for 2hPG; *P* = 0.041 for TG; *P* = 0.046 for HOMA-IR; *P* = 0.011 for visfatin; *P* = 0.042 for leptin; *P* = 0.039 for resistin, respectively), but the adiponectin level was significantly lower [(5.6 ± 3.7 vs 9.8 ± 4.8) mg/L, *P* = 0.024). The other clinical parameters were not statistically significant between the two groups (*P* > 0.05) (Table 1, Fig. 2).

Compared with the polyp-free group with prediabetes, the male proportion, average age, BMI, WHR, 2hPG, TGs, visfatin level, the distribution of current smoking, alcohol drinking, and the proportion of family history with colon cancer were significantly higher in the colonic polyps group with prediabetes (*P* = 0.000 for male; *P* = 0.029 for BMI; *P* = 0.033 for WHR; *P* = 0.035 for 2hPG; *P* = 0.046 for TG; *P* = 0.041 for visfatin; *P* < 0.001 for current smoking; *P* < 0.001 for alcohol drinking; *P* = 0.034 for family history with colon cancer, respectively), but the plasma adiponectin level was significantly lower [(5.6 ± 3.7 vs 9.2 ± 4.4) mg/L, *P* = 0.007]. The other clinical parameters were not statistically significant between the two groups (*P* > 0.05) (Table 1, Fig. 2).

### Comparison of clinical parameters between the single-polyp group and multiple-polyps group with prediabetes

**Fig. 3:**
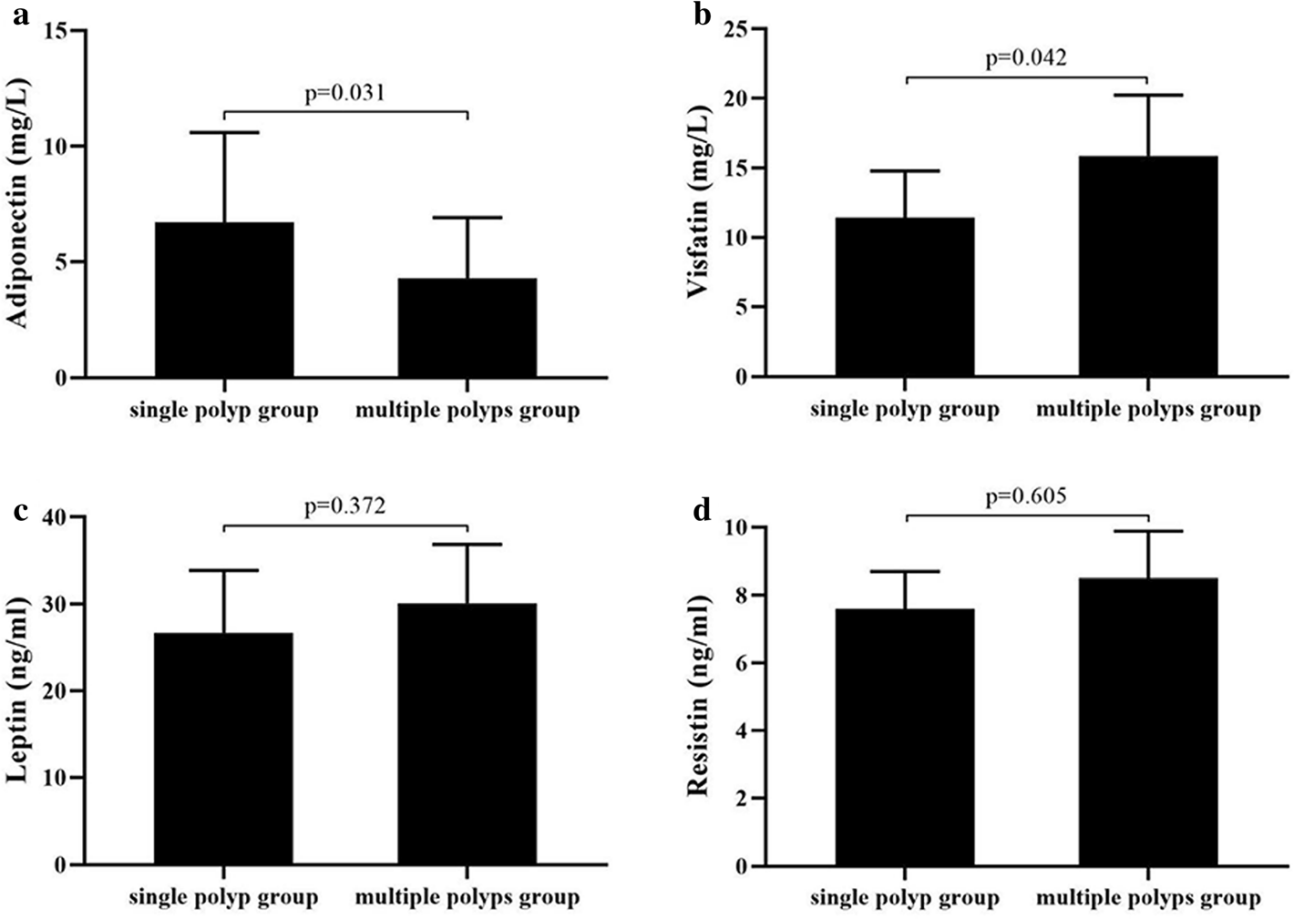
Plasma adiponectin, visfatin, leptin, and resistin levels between the single polyp group and multiple polyps group with prediabetes. (**a**) the level of plasma adiponectin was significantly lower than that in the single polyp group (*p* = 0.031), and (**b**) plasma visfatin level was higher in the multiple-polyps group than in the single-polyp group (*p* = 0.042), while (**c**) plasma leptin and (**d**) resistin levels were no significant differences between the two groups

The average age, BMI, WHR, 2hPG, FINS, HOMA-IR, plasma visfatin level, and the proportion of family history with colon cancer were significantly higher in the multiple-polyps group than in the single-polyp group (*P* = 0.025 for age; *P* = 0.038 for BMI; *P* = 0.022 for WHR; *P* < 0.001 for 2hPG; *P* = 0.034 for FINS; *P* = 0.044 for HOMA-IR; *P* = 0.042 for visfatin; *P* = 0.012 for family history with colon cancer, respectively), and the level of plasma adiponectin was significantly lower than that in the single-polyp group [(4.3 ± 2.6 vs 6.7 ± 3.9) mg/L, *P* = 0.031). No significant differences were observed in sex ratio, SBP, DBP, FPG, TG, TCH, plasma leptin and resistin levels, as well the proportion of current smoking, regular alcohol drinking, regualer exercise, and regular vegetable consumption between the two groups (*P* > 0.05). (Table 2, Fig. 3).

**Table 2:** Comparison of clinical parameters between single polyp group and multiple polyps group in prediabetes [±s, *n* (%)]

Variable	Single polyp group (*n* = 141)	Multiple polyps group (*n* = 107)	*P*-value
Sex (male) (%)	89 (63.1)	67 (52.8)	0.915
Age (year)	53.8 ± 11.2	59.7 ± 13.4	0.025
BMI (kg/m^2^)	24.2 ± 3.5	25.5 ± 3.8	0.038
WHR	0.89 ± 0.07	0.92 ± 0.09	0.022
SBP (mm Hg)	123 ± 14	125 ± 13	0.862
DBP (mm Hg)	77 ± 13	78 ± 12	0.895
FPG (mmol/L)	5.9 ± 0.5	5.9 ± 0.6	0.942
2hPG (mmol/L)	8.7 ± 1.1	10.3 ± 0.7	< 0.001
TG (mmol/L)	1.64 ± 0.89	1.69 ± 0.85	0.783
TCH (mmol/L)	5.26 ± 0.92	5.33 ± 0.71	0.821
FINS (mIU/L)	10.37 ± 5.26	17.06 ± 7.18	0.034
HOMA-IR	3.11 ± 1.52	4.56 ± 1.85	0.044
Adiponectin (mg/L)	6.7 ± 3.9	4.3 ± 2.6	0.031
Visfatin (mg/L)	11.4 ± 5.9	15.9 ± 7.5	0.042
Leptin (ng/ml)	26.7 ± 12.4	30.1 ± 11.7	0.372
Resistin (ng/ml)	7.6 ± 1.9	8.5 ± 2.4	0.605
Current smoking (%)	44 (31.2)	51 (47.7)	0.575
Regular alcohol drinking (%)	45 (31.9)	52 (48.6)	0.961
Regular exercise (%)	32 (22.7)	21 (19.6)	0.559
Regular vegetables consumption (%)	41 (29.1)	28 (26.2)	0.613
Family history of colon cancer (%)	10 (7.1)	15 (14.0)	0.012

Data are presented mean ± standard deviations or numbers (%); Differences between the two groups compared using the independent sample t-test to analyze the normally distributed continous variables, using the Mann-Whitney U test to analyze the abnormally distributed continous variables. And using *x*^2^ test to analyze the count data

### Comparison of clinical parameters between the low-risk polyps group and the high-risk polyps group with prediabetes

**Fig. 4:**
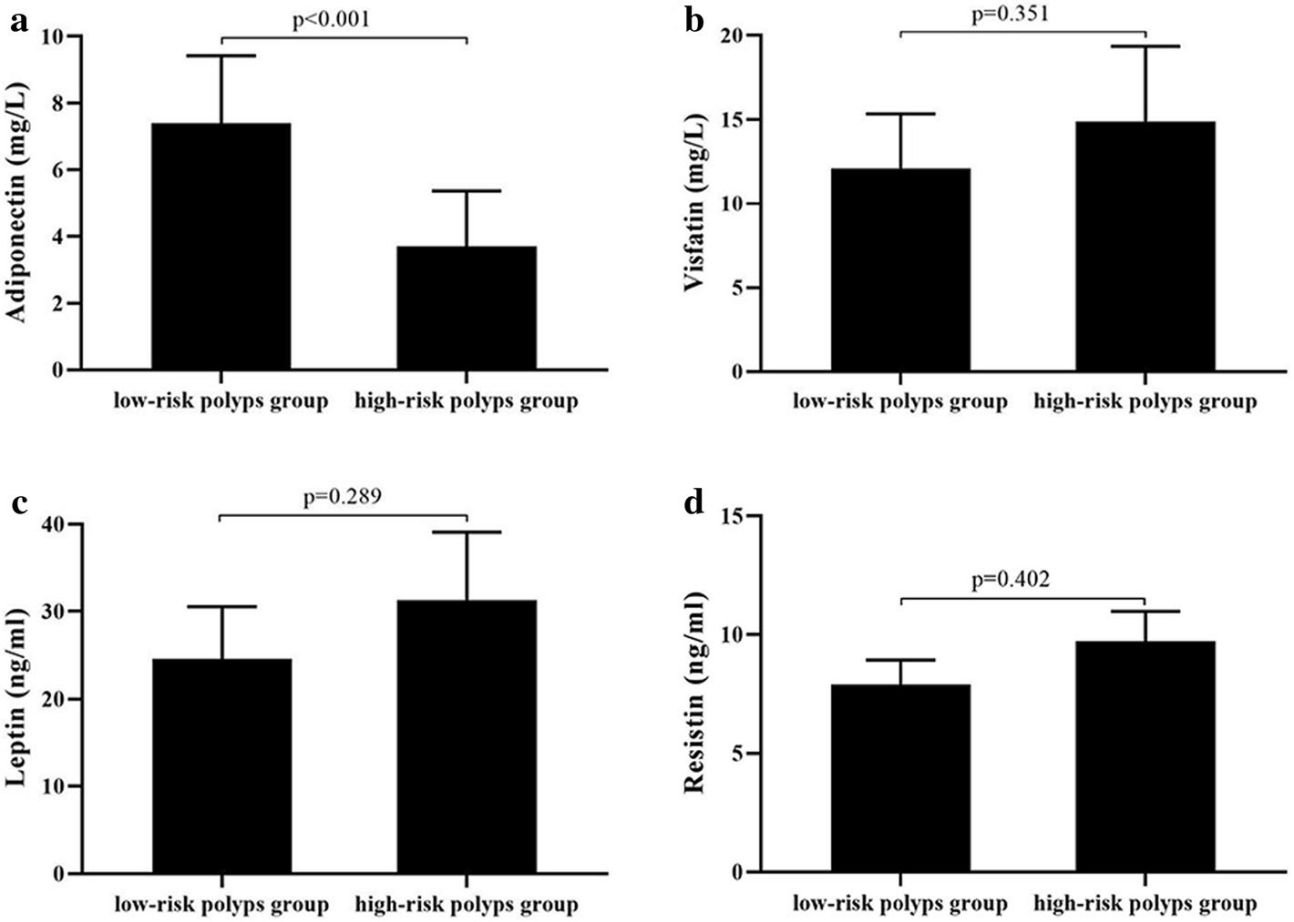
Plasma adiponectin, visfatin, leptin, and resistin levels between the low-risk polyps group and high-risk polyps group with prediabetes. (**a**) the level of plasma adiponectin was significantly lower than that in the low-risk polyps group (*p* < 0.001), while (**b**) plasma visfatin, (**c**) leptin and (**d**) resistin levels were no significant differences between the two groups

The proportion of males, average age, BMI, WHR, 2hPG, FINS, HOMA-IR, distribution of current smoking, and the proportion of family history with colon cancer in the high-risk polyps group were significantly higher than those in the low-risk polyps group (*P* = 0.029 for male; *P* = 0.012 for age; *P* = 0.046 for BMI; *P* = 0.032 for WHR; *P* < 0.001 for 2hPG; *P* = 0.047 for FINS; *P* = 0.034 for HOMA-IR; *P* = 0.023 for smoking; *P* = 0.035 for family history with colon cancer, respectively), whereas the level of plasma adiponectin was significantly lower than that in the low-risk polyps group [(3.7 ± 2.9 vs 7.4 ± 3.5) mg/L, *P* < 0.001). No significant differences were observed in SBP, DBP, FPG, TGs, TCH, plasma visfatin, leptin, and resistin levels, as well the proportion of regular alcohol drinking, regualer exercise, and regular vegetable consumption between the two groups (*P* > 0.05). (Table 3, Fig. 4).

**Table 3:** Comparison of clinical parameters between low-risk polyps group and high-risk polyps group in prediabetes [±s, *n* (%)]

Variable	Low-risk polyps group (*n* = 90)	High-risk polyps group (*n* = 158)	*P*-value
Sex (male) (%)	43 (47.8)	98 (62.5)	0.029
Age (year)	54.7 ± 10.9	58.1 ± 12.4	0.012
BMI (kg/m^2^)	23.6 ± 3.3	24.9 ± 3.6	0.046
WHR	0.88 ± 0.08	0.92 ± 0.09	0.032
SBP (mm Hg)	122 ± 16	123 ± 15	0.876
DBP (mm Hg)	76 ± 14	77 ± 12	0.913
FPG (mmol/L)	5.8 ± 0.4	5.9 ± 0.6	0.882
2hPG (mmol/L)	8.8 ± 0.9	10.3 ± 0.6	< 0.001
TG (mmol/L)	1.61 ± 0.89	1.68 ± 0.91	0.621
TCH (mmol/L)	5.02 ± 0.88	5.17 ± 0.72	0.705
FINS (mIU/L)	12.62 ± 5.93	18.98 ± 8.23	0.047
HOMA-IR	3.38 ± 1.76	5.02 ± 2.28	0.034
Adiponectin (mg/L)	7.4 ± 3.5	3.7 ± 2.9	< 0.001
Visfatin (mg/L)	12.1 ± 5.6	14.9 ± 7.7	0.351
Leptin (ng/ml)	24.6 ± 10.3	31.3 ± 13.5	0.289
Resistin (ng/ml)	7.9 ± 1.8	9.7 ± 2.2	0.402
Current smoking (%)	18 (20.0)	77 (48.7)	0.023
Regular alcohol drinking (%)	32 (35.6)	65 (41.1)	0.386
Regular exercise (%)	26 (28.9)	27 (17.1)	0.433
Regular vegetables consumption (%)	27 (30.0)	42 (26.6)	0.564
Family history of colon cancer (%)	5 (5.6)	18 (11.4)	0.035

Data are presented mean ± standard deviations or numbers (%); Differences between the two groups compared using the independent sample t-test to analyze the normally distributed continous variables, using the Mann-Whitney U test to analyze the abnormally distributed continous variables. And using *x*^2^ test to analyze the count data

### Analysis of risk factors for colonic polyps

To identify the risk factors for colonic polyps in prediabetes subjects, the multivariate unconditional logistic regression analysis was performed after adjustment for sex, age, BMI, WHR, 2hPG, TGs, adiponectin, visfatin, leptin, resistin, current smoking, alcohol drinking, family history of colon cancer to determine the risk factors for colonic polyps in prediabetes subjects. At the same time, we carried out a multivariate unconditional logistic regression analysis adjusted for age, BMI, WHR, 2hPG, FINS, HOMA-IR, adiponectin, visfatin, and family history of colon cancer to determine the risk factors for multiple colonic polyps in prediabetes subjects. In addition, in order to determine the risk factors for prediabetes-related high-risk colonic polyps, We performed a multivariate unconditional logistic regression analysis adjusted for sex, age, BMI, WHR, 2hPG, FINS, HOMA-IR, adiponectin, current smoking, and family history of colon cancer. In these analyses, sex, WHR, 2hPG, and family history of colon cancer were found to be risk factors for colonic polyps in prediabetes subjects (OR values were 1.89, 2.98, 1.81, 1.36, respectively, *P* < 0.05) (Table 4). In addition, age, WHR, and HOMA-IR were risk factors for multiple colonic polyps in prediabetes subjects (OR values were 1.09, 2.12, 1.76, respectively, *P* < 0.05) (Table 5). Moreover, age and WHR were risk factors for high risk colonic polyps in prediabetes subjects (OR values were 1.45 and 2.11, respectively, *P* < 0.05) (Table 6). Tables 4, 5 and 6 show that lower levels of plasma adiponectin was a risk factor for colonic polyps, multiple colonic polyps, and high-risk colonic polyps in prediabetes subjects (OR values were 0.67, 0.79, and 0.61, respectively, *P* < 0.05).

**Table 4:** The multiple logistic regression analysis of risk factors of colonic polyps in prediabetes

Variable	β	SE	Wald	OR	95% CI	*P*-value
Sex (male)	0.52	0.39	5.12	1.89	1.23 ~ 7.02	0.015
WHR	0.87	0.22	8.03	2.98	1.41 ~ 5.03	0.039
2hPG (mmol/L)	0.79	0.14	3.75	1.81	1.12 ~ 8.06	0.042
Family history of colon cancer	0.61	0.43	3.98	1.36	1.07 ~ 8.57	0.048
Adiponectin (mg/L)	−0.68	0.19	4.27	0.67	0.43 ~ 0.89	0.009

Multivariate unconditional logistic regression analysis adjusted for sex, age, BMI, WHR, 2hPG, TG, adiponectin, visfatin, leptin and resistin, current smoking, alcohol drinking, and family history of colon cancer

**Table 5:** The multiple logistic regression analysis of risk factors of multiple colonic polyps in preiabetes

Variable	β	SE	Wald	OR	95% CI	*P*-value
Age (year)	0.44	0.16	1.98	1.09	1.11 ~ 9.76	0.046
WHR	0.76	0.29	3.95	2.12	1.27 ~ 6.21	0.019
HOMA-IR	0.55	0.18	2.46	1.76	1.32 ~ 7.78	0.041
Adiponectin (mg/L)	−0.82	0.23	3.12	0.79	0.52 ~ 0.94	0.021

Multivariate unconditional logistic regression analysis adjusted for age, BMI, WHR, 2hPG, FINS, HOMA-IR, adiponectin, visfatin, and family history of colon cancer

**Table 6:** The multiple logistic regression analysis of risk factors of high risk colon polyps in prediabetes

Variable	β	SE	Wald	OR	95% CI	*P*-value
Age (year)	0.55	0.19	2.12	1.45	1.09 ~ 8.78	0.024
WHR	0.82	0.23	3.23	2.11	1.27 ~ 6.92	0.037
Adiponectin (mg/L)	−0.61	0.28	4.57	0.61	0.39 ~ 0.98	0.001

Multivariate unconditional logistic regression analysis adjusted for sex, age, BMI, WHR, 2hPG, FINS, HOMA-IR, adiponectin, current smoking, and family history of colon cancer

## Discussion

It is generally believed that age, poor lifestyle habits (such as lack of exercise, smoking, excessive alcohol consumption, high-fat/low-fibre diet, etc.), overweight/obesity, hyperinsulinaemia/insulin resistance, genetic susceptibility, inflammatory bowel disease, etc. are closely related to the development of colonic polyps [16–18]. In the present study, we revealed that age and family history of colon cancer were related to the incidence of colonic polyps in the population with normal glucose tolerance, while in prediabetes patients, we found that sex (male), age, BMI, WHR, 2hPG, TGs, the distribution of current smoking and alcohol drinking, and family history of colon cancer were associated with the development of colonic polyps. Further analysis showed that sex (male), WHR, 2hPG, and family history of colon cancer were risk factors for the development of colonic polyps in prediabetes patients. These results are basically consistent with previous research findings [7, 14, 19]. In addition, we demonstrated that plasma visfatin and adiponectin levels but not plasma leptin and resistin levels are closely associated with the development of colonic polyps in prediabetes subjects. The multivariate logistic regression analysis suggested that lower levels of plasma adiponectin was a risk factor for colonic polyps. To the best of our knowledge, this is the first study to evaluate the role of adiponectin, visfatin, leptin, and resistin in the development of colonic polyps in prediabetes subjects.

Further analysis showed that factors associated with the incidence of multiple polyps in the colons of prediabetes subjects included age, BMI, WHR, HOMA-IR, 2hPG, TGs, and FINS levels. The multivariate regression analysis found that age, WHR, and HOMA-IR were risk factors for multiple polyps in the colon. Similarly, in the present study, we revealed that factors associated with high-risk colonic polyps in prediabetes subjects included sex (male), age, BMI, WHR, HOMA-IR, 2hPG, and FINS levels. The multivariate regression analysis found that age and WHR were risk factors for high-risk polyps in the colon. Studies have confirmed that WHR and HOMA-IR play important roles in the development of cardiovascular disease in diabetic and non-diabetic patients [20, 21]. And the above results of this study suggest that more attention should be paid to the presence of multiple colonic polyps in prediabetes subjects who are men who suffer from abdominal obesity and insulin resistance. It is worth noting that the risk of malignant polyps in the colon needs to be monitored in older prediabetes patients with abdominal obesity.

Adiponectin, leptin, visfatin, and resistin are all adipokines secreted by white adipose tissue that have different physiological effects. In recent years, studies have found that these four adipokines may play an important role in the mechanism linking obesity and colon tumour development [9, 12, 22]. The data from the present study showed that the plasma adiponectin levels significantly decreased and the visfatin levels significantly increased in subjects with normal glucose tolerance with colonic polyps compared with those with normal glucose tolerance without colonic polyps. Similarly, in prediabetes subjects, plasma adiponectin levels were significantly lower and visfatin levels were significantly higher in the colonic polyps group and multiple-polyps group than in the polyp-free group and single-polyp group, respectively. In addition, plasma adiponectin levels were lower in the high-risk polyps group than in the low-risk polyps group in prediabetes subjects. Nevertheless, no significant differences were found in plasma visfatin levels between the high-risk polyps group and the low-risk polyps group. Multivariate regression analysis found that the incidence of colonic polyps, multiple polyps and high-risk colonic polyps were closely related to plasma adiponectin levels. This finding suggested that adiponectin might play a role in the pathogenesis and malignant progression of colonic polyps in prediabetes patients, whereas visfatin might be involved in only the development of colonic polyps and have a limited impact on the progression of the malignant transformation of colonic polyps.

It is currently believed that adiponectin has indirect and direct effects on the pathogenesis of colon cancer [23]. The indirect effects include the improvement of insulin resistance and hyperinsulinaemia, the inhibition of tumour angiogenesis, and the inhibition of mutations in inflammatory proto-oncogenes (such as the K-ras gene, etc.). The direct action mainly involves the anti-proliferation and pro-apoptosis effects of adiponectin. Although the molecular mechanism linking visfatin and colon cancer is still unclear, studies have shown that visfatin has an insulin-like effects binding with insulin receptors to promote proliferation of cancer cells. Huang et al. revealed that visfatin increased stromal cell-derived factor-1 (SDF-1) expression in colorectal cancer DLD-1 cells, which was mediated by β1 integrin [24]. It is well known that SDF-1 may play an important role in colorectal cancer development and progression.

In addition, the present study showed no significant difference in plasma leptin and resistin levels between the colonic polyps group and the polyp-free group in both normal glucose tolerance subjects and prediabetes patients. Similarly, there were no significant differences in plasma leptin and resistin levels between the single-polyp group and the multiple-polyps group or between the low-risk polyps group and the high-risk polyps group. These findings suggest that leptin and resistin may not have been the main influencing factors in the development of colonic polyps in the subjects in the present study. To date, many clinical studies on the correlation between leptin and colon cancer have been reported. The results are inconsistent and show racial differences. Cong et al. found that serum leptin levels both in men and women with colon cancer were significantly higher than those in the normal control group in a Chinese population, which is inconsistent with the results of our study [25]. This discrepancy may be due to a difference in glucose metabolism status and the degree of malignancy of the colon tumours. Kumor et al. found that serum resistin levels in patients with colorectal adenomatous polyps were significantly higher than those in the polyp-free group, which is also inconsistent with the results of the present study [26]. This discrepancy may also be attributable to racial differences and differences in the glucose metabolism status of the subjects.

The main shortcomings of this study are as follows: (1) the study analysed only prediabetes patients over 40 years old at high risk for colon cancer, so the conclusions are difficult to extrapolate to the overall prediabetes population; (2) the present study was a cross-sectional analysis, so it was difficult to determine the causal relationship between colonic polyps and the related influencing factors; (3) the cases are composed of not only prediabetes, but also “high-risk of colon cancer by the screening strategies”, so the findings presented here might overestimate the odds ratios of the risk of colonic polyps in the general prediabetes population; (4) in the present study, we merely considered two (sex and age) matched factors between controls (*N* = 108) and the prediabetes subjects (*N* = 468), which might result in additional bias in our analyses [27]; and (5) it has been revealed that several factors, like C-peptide, insulin-like growth factor 1/2, insulin-like growth factor binding protein 1/2, are linked to development and progression of colon carcinoma. Regrettably, we did not detect these indicators, as we measured only plasma adiponectin, visfatin, leptin, and resistin. Therefore, our results need to be confirmed with more studies in view of these shortcomings. A large-scale, multicentre, prospective cohort study is needed to clarify the causal relationship between colonic polyps and related influencing factors.

## Conclusion

Among prediabetes subjects, sex (male), WHR, and 2hPG were risk factors for colonic polyps. Age and WHR were positively correlated with the incidence of multiple polyps and high-risk polyps in the colon. Plasma adiponectin levels are inversely associated with colonic polyps, including multiple polyps and high-risk polyps in prediabetes subjects. Adiponectin may be involved in the development and progression of colon tumours in prediabetes subjects. And further studies are need to evaluate the potential increase of adiponectin levels in the reduction of colonic polys risk.

## Data Availability

The datasets used and/or analysed during the current study are available from the corresponding author on reasonable request. Inquiries for data access may be sent to the following e-mail address: chmw1@163.com.

## Abbreviations

SBP: Systolic blood pressure
DBP: Diastolic blood pressure
BMI: Body mass index
WHR: Waist:hip ratio
TCH: Total cholesterol
TGs: Triglycerides
HOMA-IR: Homeostatic model assessment of insulin resistance
FPG: Fasting plasma glucose
2hPG: Postprandial 2 h plasma glucose
FINS: Fasting insulin

## References

[1] Aroda VR, Ratner R (2008). Approach to the patient with prediabetes. J Clin Endocrinol Metab.

[2] Antonino DP, Sarah M, Francesca U (2017). HbA1c identifies subjects with prediabetes and subclinical left ventricular diastolic dysfunction. J Clin Endocrinol Metab.

[3] Scicali R, Giral P, Gallo A (2016). HbA1c increase is associated with higher coronary and peripheral atherosclerotic burden in non diabetic patients. Atherosclerosis..

[4] Zhou XH, Qiao Q, Zethelius B (2010). Diabetes, prediabetes and cancer mortality. Diabetologia..

[5] Ferlay J, Shin HR, Bray F, Forman D, Mathers C, Parkin DM (2010). Estimates of worldwide burden of cancer in 2008: GLOBOCAN 2008. Int J Cancer.

[6] Cha JM, Lee JI, Joo KR, Shin HP, Jeun JW, Lim JU (2013). Prediabetes is associated with a high-risk colorectal adenoma. Dig Dis Sci.

[7] Dong Y, Zhou J, Zhu Y, et al. Abdominal obesity and colorectal cancer risk: systematic review and meta-analysis of prospective studies. Biosci Rep. 2017;37(6):BSR20170945. 10.1042/BSR20170945 PMC5725611 29026008

[8] Aleksandrova K, Nimptsch K, Pischon T (2013). Influence of obesity and related metabolic alterations on colorectal Cancer risk. Curr Nutr Rep.

[9] Kim JH, Lim YJ, Kim YH (2007). Is metabolic syndrome a risk factor for colorectal adenoma?. Cancer Epidemiol Biomark Prev.

[10] Comstock SS, Hortos K, Kovan B, McCaskey S, Pathak DR, Fenton JI (2014). Adipokines and obesity are associated with colorectal polyps in adult males: a cross-sectional study. PLoS One.

[11] Aleksandrova K, Nimptsch K, Pischon T (2013). Obesity and colorectal cancer. Front Biosci (Elite Ed).

[12] Joshi RK, Lee SA (2014). Obesity related adipokines and colorectal cancer: a review and meta-analysis. Asian Pac J Cancer Prev.

[13] Chen M, Wang Y, Li Y (2016). Association of plasma visfatin with risk of colorectal cancer: an observational study of Chinese patients. Asia Pac J Clin Oncol.

[14] Chen MW, Zhao XT, Zhao LL (2015). Analysis of population screening results for colorectal cancer and the risk factors of colorectal adenoma in prediabetes in Hefei communities. Chin J Endocrinol and Metab.

[15] Chen MW, Ye S, Zhao LL (2012). Association of plasma total and high-molecular-weight adiponectin with risk of colorectal cancer: an observational study in Chinese male. Med Oncol.

[16] Lieberman DA, Prindiville S, Weiss DG, Willett W, VA Cooperative study group 380. Risk factors for advanced colonic neoplasia and hyperplastic polyps in asymptomatic individuals. JAMA. 2003;290(22):2959–2967. 10.1001/jama.290.22.2959 14665657

[17] Levin B, Lieberman DA, McFarland B (2008). Screening and surveillance for the early detection of colorectal cancer and adenomatous polyps, 2008: A joint guideline from the American Cancer Society, the US Multi-Society Task Force on Colorectal Cancer, and the American College of Radiology. Gastroenterology..

[18] Jochem C, Leitzmann M (2016). Obesity and colorectal cancer. Recent Results Cancer Res.

[19] Haggar FA, Boushey RP (2009). Colorectal cancer epidemiology: incidence, mortality, survival, and risk factors. Clin Colon Rectal Surg.

[20] Adeva-Andany MM, Martínez-Rodríguez J, González-Lucán M, Fernández-Fernández C, Castro-Quintela E (2019). Insulin resistance is a cardiovascular risk factor in humans. Diabetes Metab Syndr.

[21] Scicali R, Rosenbaum D, Di Pino A (2018). An increased waist-to-hip ratio is a key determinant of atherosclerotic burden in overweight subjects. Acta Diabetol.

[22] Fujisawa T, Endo H, Tomimoto A (2008). Adiponectin suppresses colorectal carcinogenesis under the high-fat diet condition. Gut..

[23] Muppala S, Konduru SKP, Merchant N (2017). Adiponectin: Its role in obesity-associated colon and prostate cancers. Crit Rev Oncol Hematol.

[24] Huang WS, Chen CN, Sze CI, Teng CC (2013). Visfatin induces stromal cell-derived factor-1 expression by β1 integrin signaling in colorectal cancer cells. J Cell Physiol.

[25] Cong JC, Dai XW, Shen MY, Wang JJ, Chen CS, Zhang H (2009). Expression of obesity hormone leptin in human colorectal cancer. Chin J Cancer Res.

[26] Kumor A, Daniel P, Pietruczuk M, Malecka-Panas E (2009). Serum leptin, adiponectin, and resistin concentration in colorectal adenoma and carcinoma (CC) patients. Int J Colorectal Dis.

[27] de Graaf MA, Jager KJ, Zoccali C, Dekker FW (2011). Matching, an appealing method to avoid confounding?. Nephron Clin Pract.

